# Burden of Five Musculoskeletal Disorders in ASEAN Countries, 1990–2023: A Global Burden of Disease Study 2023 Analysis

**DOI:** 10.3390/jcm15197476

**Published:** 2026-09-25

**Authors:** Keith Pardillada Belangoy, Yoshito Nishimura, Ko Harada, Hideharu Hagiya, Quynh Thi Vu, Hanane Ouddoud, Judah Israel Ong Lescano, Elizabeth Tan, Michio Yamamoto, Tatsuaki Takeda, Naohiro Iwata, Toshihiro Koyama, Yoshito Zamami

**Affiliations:** 1 Department of Health Data Science, Graduate School of Medicine, Dentistry and Pharmaceutical Sciences, Okayama University, Okayama 7008558, Japan; 2 Department of Medicine, Mayo Clinic, Rochester, MN 55905, USA; 3 Division of General Internal Medicine, Washington University School of Medicine, St. Louis, MO 63110, USA; 4 Department of Infectious Diseases, Okayama University Hospital, Okayama 7008558, Japan; 5 Faculty of Pharmacy, Haiphong University of Medicine and Pharmacy, Haiphong 180000, Vietnam; 6 Department of Pharmacy, University of San Carlos, Cebu City 6000, Philippines; 7 Graduate School of Human Sciences, The University of Osaka, Osaka 5650871, Japan; 8 RIKEN Center for Advanced Intelligence Project, Tokyo 1030027, Japan; 9 Data Science and AI Innovation Research Promotion Center, Shiga University, Hikone 5228522, Japan; 10 Department of Education and Research Center for Clinical Pharmacy, Faculty of Pharmaceutical Sciences, Okayama University, Okayama 7008558, Japan; 11 Department of Pharmacy, Okayama University Hospital, Okayama 7008558, Japan

**Keywords:** musculoskeletal disorders, ASEAN, osteoarthritis, rheumatoid arthritis, gout, low back pain, neck pain, prevalence, disability-adjusted life years

## Abstract

**Background/Objectives:** Five musculoskeletal disorders (MSDs), including gout, low back pain, neck pain, osteoarthritis, and rheumatoid arthritis represent a growing public health burden; however, evidence from the Association of Southeast Asian Nations (ASEAN) remains limited. This study aimed to assess the burden of these five MSDs across ten ASEAN countries. **Methods:** Using data from the Global Burden of Disease Study 2023, we estimated the burden using prevalence and disability-adjusted life years (DALYs). National trends from 1990 to 2023 were assessed using average annual percent change (AAPC). **Results:** From 1990 to 2023, the absolute numbers of prevalent cases and absolute numbers of DALYs increased, with low back pain and osteoarthritis accounting for large shares across the ten countries. Country-level patterns differed between absolute numbers and age-standardised rates of the five MSDs. In 2023, age-specific absolute numbers of prevalent cases and absolute numbers of DALYs increased with age before declining in older age groups, with peak ages varying across the five MSDs. Across the five MSDs, age-standardised prevalence rates were generally higher among females, while gout showed higher rates among males. Age-standardised prevalence rates showed generally modest positive AAPCs for osteoarthritis, rheumatoid arthritis, and gout, whereas low back pain and neck pain showed decreasing trends in most countries. **Conclusions:** The increasing burden in absolute numbers of the five MSDs across the ten ASEAN countries, together with persistent sex disparities, differences in age-specific patterns, variations in age-standardised trends, and considerable country-level heterogeneity, highlights the need for continued attention to the burden of the five MSDs in the region.

## 1. Introduction

In 2021, musculoskeletal disorders (MSDs) accounted for an estimated 1.6 billion prevalent cases worldwide [1]. They also affect individuals across the life course, from adolescence to older adulthood, underscoring their relevance beyond ageing populations alone [2]. MSDs, including gout, low back pain, neck pain, osteoarthritis, and rheumatoid arthritis, contribute considerably to this burden through their high prevalence and associated disability [1,2,3]. The burden of disability attributable to low back pain and neck pain remains substantial, particularly among working-age populations [4]. Increasing burdens of osteoarthritis, gout, and rheumatoid arthritis have been reported, highlighting the influence of population ageing and changing disease patterns [1,2].

The economic and social impact of MSDs is often underestimated because of their low mortality rates [2,4,5]. These disorders restrict daily functioning and work participation, leading to early retirement and greater reliance on social support [6]. MSDs are among the leading contributors to rehabilitation needs worldwide because of their substantial impact on quality of life. Estimates from the Global Burden of Disease Study 2019 (GBD 2019) further indicate that Southeast Asia has one of the highest global needs for rehabilitation services, with MSDs contributing substantially to this demand [7]. In addition, rapid socioeconomic development and population growth in the Association of Southeast Asian Nations (ASEAN) have driven increasing emphasis on health promotion, occupational health, and active ageing [8,9]. These priorities further highlight the importance of addressing MSDs in the region, given their considerable public health impact.

Previous epidemiological studies have identified gout, low back pain, neck pain, osteoarthritis, and rheumatoid arthritis among the leading contributors to the burden of MSDs [1,3,6]. Despite differences in their underlying pathology and clinical presentation, these five MSDs share clinical consequences that may lead to disability and reduced quality of life, placing a substantial burden on individuals and health systems [1,4,10,11,12]. Understanding the distribution and trends in their burden is therefore important for guiding attention to prevention and intervention strategies. Although the burden of MSDs has been widely reported, no comprehensive analysis has examined the burden of the five MSDs across the ten ASEAN countries. Using data from the GBD 2023, we assessed the burden of these five MSDs from 1990 to 2023 and examined age- and sex-specific burden patterns and temporal trends. These findings may support burden monitoring and provide evidence relevant to the development of programmes addressing the five MSDs in the ten ASEAN countries.

## 2. Materials & Methods

### 2.1. Data Source

This study used data from the GBD 2023, a comprehensive assessment providing estimates for 204 countries and territories from 1990 to 2023 across 375 diseases and injuries and 88 modifiable risk factors [13]. We extracted data from the Global Health Exchange tool for the five selected MSDs [14]. Extracted data included prevalence and disability-adjusted life years (DALYs) reported as absolute numbers and age-standardised rates per 100,000 population, with corresponding 95% uncertainty intervals (UIs), by age, sex and location.

### 2.2. Selection of the Five MSDs

The five MSDs included in this analysis were gout, low back pain, neck pain, osteoarthritis, and rheumatoid arthritis. These conditions were selected based on their established contribution to the MSD burden and their changing burden patterns over time, as reported in the previous epidemiological literature [1,3,6]. These five MSDs were represented as distinct conditions in the GBD 2023, enabling a focused assessment of their prevalence, DALYs, and temporal trends across the ten ASEAN countries. However, the findings do not represent the overall burden of all MSDs included in GBD 2023 across the ten ASEAN countries.

### 2.3. Case Definition

The International Classification of Diseases, Tenth Revision (ICD-10) coding system was used to define the following conditions: gout (M10), low back pain (M54.3, M54.4, and M54.5), neck pain (M54.2), osteoarthritis (M16 and M17), and rheumatoid arthritis (M05, M06, and M07) [15].

### 2.4. Measures

We reported prevalence and DALYs between 1990 and 2023 (absolute numbers and age-standardised rates per 100,000 population), with 95% UIs. Analyses were conducted using GBD 5-year age groups for populations aged 5 years and older; age groups younger than 5 years were excluded because all five MSDs had zero estimates.

Prevalence was defined as the proportion of the population affected by a specific disease at a given time point. DALYs were calculated as the sum of years lived with disability (YLDs; nonfatal health loss) and years of life lost (YLLs; premature mortality). Age-standardised rates from the GBD 2023 were used to account for differences in age structure across populations.

### 2.5. Ten ASEAN Countries

The ten ASEAN countries included in this study were Brunei, Cambodia, Indonesia, Laos, Malaysia, Myanmar, the Philippines, Singapore, Thailand, and Vietnam. Data were retrieved individually for each country using the GBD 2023 Global Health Exchange tool.

### 2.6. Age-Specific and Relative Distribution of the Five MSDs

To describe the age-specific burden in 2023, absolute numbers of prevalent cases and absolute numbers of DALYs were summed across the ten ASEAN countries for each age group and each of the five MSDs. To describe the relative distribution of prevalent cases across the five MSDs, the proportion of prevalent cases was calculated for each country and year by dividing the absolute number of prevalent cases for a given MSD by the total absolute number of prevalent cases across the five MSDs and multiplying by 100. Proportions were calculated separately for each of the ten ASEAN countries in 1990 and 2023.

### 2.7. Statistical Analyses

Temporal trends in annual age-standardised prevalence point estimates for each of the five MSDs were assessed using Joinpoint regression for each country, using annual estimates from 1990 to 2023. A log linear model was fitted to the annual age-standardised prevalence point estimates. Models allowed for 0 to 6 joinpoints and were selected based on the minimum weighted Bayesian Information Criterion (WBIC), assuming uncorrelated errors, without permutation testing for model selection. The annual percent change (APC) represents the annual rate of change in the age-standardised prevalence rate within each segment of the fitted model. The average annual percent change (AAPC) is a summary measure of the temporal trend over a pre-specified fixed interval and was calculated as the weighted average of segment-specific APCs, with weights proportional to the segment length, to summarise the overall trend from 1990 to 2023. The corresponding 95% confidence intervals (CIs) for APCs and AAPCs were estimated using the empirical quantile method with 5001 resamples in the Joinpoint Regression Program. Statistical significance was defined as *p* < 0.05, and the 95% CI for the AAPC did not include zero [16,17]. The analysis used annual point estimates only; the GBD 95% UIs were not used as weights or otherwise incorporated into the Joinpoint analysis.

Statistical analyses were performed using Joinpoint Regression Program (version 5.4.0.0, April 2025; Statistical Research and Applications Branch, National Cancer Institute) and R software version 4.4.3 for data processing and visualisation.

## 3. Results

From 1990 to 2023, the absolute numbers of prevalent cases and absolute numbers of DALYs for the five MSDs increased across ten ASEAN countries and showed comparable patterns. Indonesia, Thailand, Vietnam, and the Philippines had the largest absolute numbers of prevalent cases, whereas age-standardised prevalence rates for most MSDs were higher in Singapore and Brunei. The age-standardised prevalence rates and age-standardised DALY rates of osteoarthritis, gout and rheumatoid arthritis increased over time. In contrast, low back pain and neck pain showed a decline in age-standardised prevalence rates in most countries, although both were dominant contributors to the absolute numbers of DALYs.

### 3.1. Age-Specific Absolute Numbers of Prevalent Cases and DALYs

In 2023, low back pain had the largest absolute number of prevalent cases among the five MSDs, with the absolute number of prevalent cases increasing from 5 to 9 years and peaking at 50–54 years (Figure 1). The absolute number of prevalent cases of osteoarthritis increased sharply from age 50–54 years, peaked at 60–64 years, and declined thereafter until ≥95 years, although it was higher than that of low back pain at older ages. The absolute number of prevalent cases of gout increased from age 25–29 and peaked at 60–64 years, while those of neck pain and rheumatoid arthritis peaked at 45–49 years and 55–59 years, respectively. Age-specific absolute numbers of DALYs followed a similar pattern, increasing with age before declining in older age groups, with low back pain contributing the largest absolute numbers of DALYs across all age groups (Figure 2). The absolute numbers of DALYs due to low back pain, osteoarthritis, and neck pain peaked in the same age groups as their absolute numbers of prevalent cases. In contrast, the absolute number of DALYs due to gout peaked at 55–59 years, while those due to rheumatoid arthritis peaked at 60–64 years.

### 3.2. Sex-Specific Age-Standardised Prevalence Rates

Country-level sex-specific age-standardised prevalence rates showed comparable patterns across countries in 2023 (Figure 3 and Table S1). Gout was the only condition with substantially higher age-standardised prevalence rates in males, with rates approximately three times higher than those observed in females, whereas the other four MSDs showed higher rates among females.

### 3.3. Country-Specific Prevalence and DALYs

Tables S2.1–2.5 show the absolute numbers and age-standardised rates of prevalence and DALYs for the five MSDs in 1990 and 2023, along with the corresponding percentage changes. By 2023, Indonesia consistently had the largest absolute numbers of prevalent cases and absolute numbers of DALYs across all five MSDs, with low back pain accounting for the largest absolute number of prevalent cases (18.2 million [95% UI 15.8–20.4 million]) and absolute number of DALYs (2.06 million [95% UI 1.4–2.7 million]). In contrast, age-standardised prevalence rates and age-standardised DALY rates declined for low back pain and neck pain in most countries between 1990 and 2023. In 2023, Singapore and Brunei recorded the highest age-standardised prevalence rates for most of the five MSDs, with age-standardised prevalence rates for low back pain, osteoarthritis, and rheumatoid arthritis ranging from approximately 7400–8500, 6900–8800, and 150–230 per 100,000 population, respectively. Similar patterns were observed in age-standardised DALY rates for low back pain, osteoarthritis, and rheumatoid arthritis, ranging from approximately 839–964, 320–432, and 21–45 per 100,000 population, respectively. Likewise, age-standardised prevalence rates and age-standardised DALY rates for gout were highest in Singapore, Brunei, Malaysia, Thailand, and Indonesia, whereas those for neck pain were highest in Indonesia and the Philippines.

Overall, the percentage changes in age-standardised prevalence rates and age-standardised DALY rates between 1990 and 2023 exceeded the global average in most ASEAN countries. Rheumatoid arthritis showed the greatest increase in age-standardised prevalence rates, with Vietnam recording the largest percentage increase (70.4%), more than five times the global percentage change. Similar patterns were observed for age-standardised DALY rates, with Vietnam also recording the largest percentage increase (42.9%). Singapore and Malaysia experienced the largest percentage increases in gout, with both age-standardised prevalence rates and age-standardised DALY rates increasing by more than 26%. Likewise, percentage changes in age-standardised prevalence rates and age-standardised DALY rates for osteoarthritis showed substantial increases across the ten ASEAN countries, whereas age-standardised prevalence rates and age-standardised DALY rates for low back pain and neck pain showed modest declines in most countries.

### 3.4. Relative Distribution of Prevalent Cases of the Five MSDs

Based on the absolute numbers of prevalent cases, low back pain and osteoarthritis accounted for the largest shares across the five MSDs in the ten ASEAN countries in both 1990 and 2023, although the proportion of cases attributable to low back pain declined over time (Figure 4 and Table S3). In 2023, osteoarthritis represented the largest proportion of prevalent cases among the five MSDs in Singapore (50.69%), followed by Thailand (47.19%), Malaysia (41.04%), Brunei (40.88%) and Laos (32.96%). The proportion of low back pain cases was highest in Cambodia, Laos, Brunei, the Philippines, Vietnam, and Indonesia, ranging from 41% to 45%, and lowest in Thailand (32.89%). The highest proportion of neck pain was observed in the Philippines (18.23%) and the lowest in Singapore (9.33%). Gout accounted for the highest proportion of cases in Thailand (5.16%) and the lowest in Brunei (3.77%). The proportion of rheumatoid arthritis cases ranged from 0.33% to 1.16% in Indonesia and Brunei, respectively.

### 3.5. Temporal Trends

Figure 5 shows the AAPC of age-standardised prevalence rates for the five MSDs for each country from 1990 to 2023. Among the five MSDs, rheumatoid arthritis showed the highest, although modest, increase, with an AAPC of 1.64% (95% CI 1.63–1.67) in Vietnam, whereas the Philippines was the only country to show a decrease (−0.30% [95% CI −0.31 to −0.28]). Gout and osteoarthritis showed modest increases across the ten countries, with AAPCs ranging from 0.40% to 0.71% for gout and 0.07% to 0.60% for osteoarthritis. In contrast, low back pain showed a modest increase only in Thailand (0.12% [95% CI 0.11–0.13]), whereas neck pain decreased in most countries.

## 4. Discussion

Our comprehensive assessment of the burden of the five MSDs in ten ASEAN countries from 1990 to 2023 showed an overall increase in the absolute numbers of prevalent cases and absolute numbers of DALYs, whereas trends in age-standardised prevalence rates differed by condition and country. Broadly similar patterns and consistent sex-specific differences in age-standardised prevalence rates were observed. Low back pain, osteoarthritis, and neck pain were the leading contributors to overall absolute numbers of prevalent cases and absolute numbers of DALYs. Although gout and rheumatoid arthritis accounted for fewer cases, both showed increases in age-standardised prevalence rates over the study period. Overall, these findings highlight the importance of country-specific strategies for addressing the distinct burden and trends of the five MSDs.

The increasing absolute numbers of prevalent cases and absolute numbers of DALYs observed may be related to the growing prevalence of degenerative and inflammatory conditions in ageing populations [10,11,18]. The differences in age-standardised prevalence rates between sexes highlight the importance of incorporating sex-specific considerations into prevention and management strategies for the five MSDs. In our analysis, age-standardised prevalence rates were generally higher among females across the five MSDs, except for gout, which showed higher rates among males. Although our analysis did not assess factors underlying these sex differences, previous evidence identified high body mass index (BMI) and smoking as relevant factors [2,3,6,19]. Greater emphasis on health education and supportive environments for physical activity may therefore be beneficial. Although these priorities align with the ASEAN Development Agenda, the extent of their implementation likely varies across member states.

Declining age-standardised prevalence rates of low back pain and neck pain were observed, though the reasons for these declines cannot be determined from the present analysis. However, previous studies have identified changes in occupational exposures and management practices as potential contributors. For example, several studies have reported that greater MSD risk awareness and self-management education are associated with improved MSD-related outcomes [20,21,22]. Nevertheless, both conditions continue to contribute substantially to disability burden [4,6,23]. Additionally, several studies have reported elevated MSD risk among workers engaged in informal and physically demanding occupations (e.g., farmers, street vendors, public drivers, and traffic enforcers) [24,25]. Given the widespread nature of informal employment across the ten ASEAN countries, occupational vulnerability may represent an important contextual factor influencing the burden of the five MSDs. Community-based awareness and self-management initiatives may therefore be important for working-age populations, particularly those outside formal workplace settings.

The increasing absolute burden observed in this study may pose additional challenges for healthcare systems in the ten ASEAN countries, particularly given the substantial rehabilitation needs associated with these conditions [7,26]. Moreover, previous studies have suggested that gaps in promotive and preventive efforts may contribute to the growing disability burden associated with MSDs, as many patients seek care during more advanced stages of disease [19]. Although improvements in diagnostic practices may have enhanced the recognition of some MSDs [1], delayed presentation remains a challenge in many settings. Additionally, in many ASEAN countries, priorities may be focused on the growing burden of major noncommunicable and infectious diseases [8,27,28,29], potentially limiting attention toward MSDs. Nevertheless, public health and preventive approaches remain important components of a comprehensive approach to MSD care.

Studies have consistently emphasised that strengthening health data systems and disease surveillance may clarify understanding of how health conditions are prioritised, thereby supporting policymaking, health system planning, and patient care across the region [28,30]. Given the substantial resources required and the multiple competing priorities faced by many developing countries, addressing this burden remains challenging. Nevertheless, our findings provide a regional- and country-level evidence base to inform burden monitoring and support the development of programmes aimed at reducing the health burden of the five MSDs.

### Limitations

This study relied on GBD modelled estimates that depend on data availability and the quality of underlying data, which may vary across the ten ASEAN countries. In settings where primary health and surveillance data are sparse, greater uncertainty may arise from model extrapolation, which should be considered when interpreting cross-country comparisons. Differences in diagnostic practices and healthcare access may also affect the comparability of estimates across countries. As a population-level descriptive analysis, this study describes patterns and temporal trends but cannot establish causality or determine the factors underlying observed changes. In addition, only five MSDs were included: gout, low back pain, neck pain, osteoarthritis, and rheumatoid arthritis. Therefore, the findings do not represent the overall burden of all MSDs and should be interpreted as reflecting the burden of these five MSDs. However, because the analysis used condition-specific prevalence estimates, individuals affected by more than one of the five MSDs could not be identified, and the estimates should not be interpreted as representing unique individuals or mutually exclusive conditions. Comorbidity between the five MSDs may therefore contribute to overlap in the observed burden. The analysis also did not capture subnational variation, disease severity, or differences in access to care. For age-specific analyses based on absolute numbers, regional totals are influenced by differences in population size across the ten countries, particularly Indonesia, and therefore should not be interpreted as direct comparisons of population-level risk. Finally, temporal trends were assessed using Joinpoint regression of annual GBD point estimates. Because uncertainty intervals were not incorporated into the Joinpoint analysis, the resulting AAPCs should be interpreted cautiously as statistical descriptions of trends in the modelled estimates and not as evidence of causal changes in disease burden.

## 5. Conclusions

Despite broadly comparable patterns across the region, the five MSDs continued to represent a substantial burden, with considerable heterogeneity observed between countries. The absolute numbers of prevalent cases and absolute numbers of DALYs increased across the five MSDs in the ten ASEAN countries from 1990 to 2023. Age-standardised prevalence rates increased for osteoarthritis, rheumatoid arthritis, and gout, while rates for low back pain and neck pain declined in most countries. Differences were observed across the five MSDs in age-specific absolute numbers of prevalent cases and absolute numbers of DALYs, with age peaks varying between conditions. Sex differences were also observed across the five MSDs, with age-standardised prevalence rates generally higher among females except for gout. These findings highlight the need for coordinated policies alongside approaches tailored to the distinct burden patterns of the five MSDs and their country-level and sex-specific differences. Future research should focus on integrating detailed clinical and risk factor data to identify drivers of change in the burden of the five MSDs across the ten ASEAN countries.

## Figures and Tables

**Figure 1:**
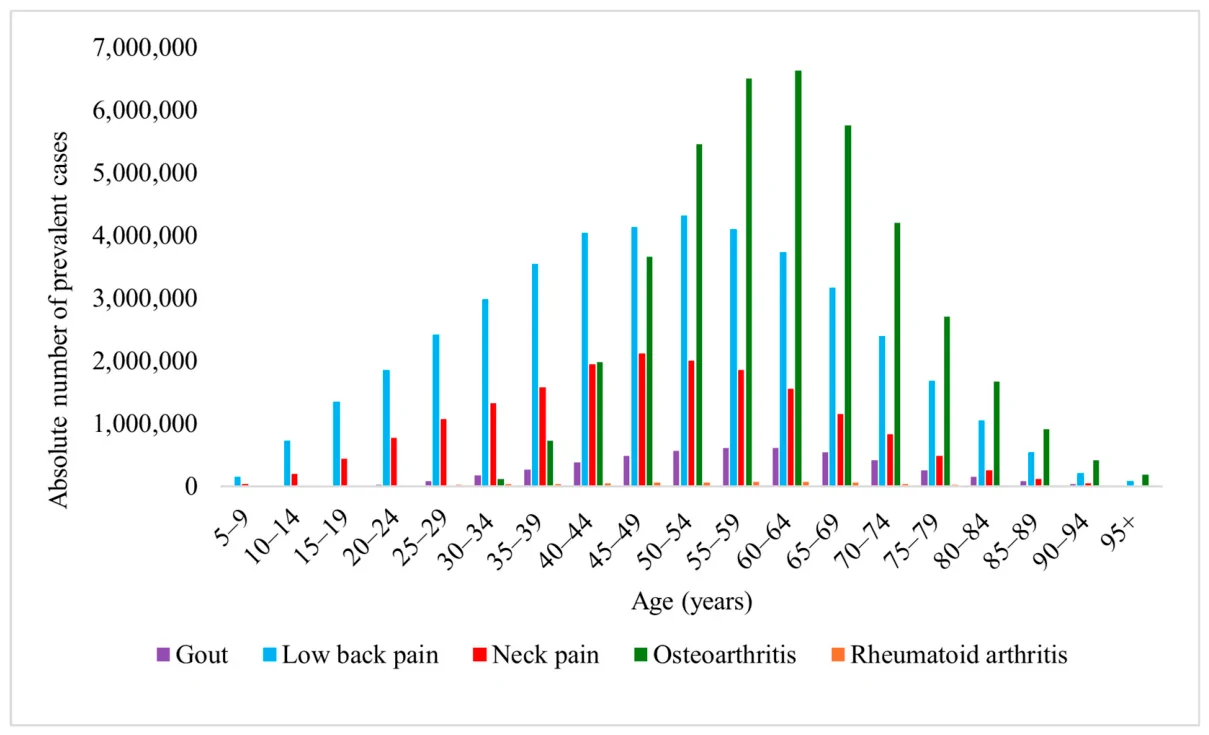
Age-specific absolute numbers of prevalent cases of the five musculoskeletal disorders across the ten ASEAN countries, 2023. Prevalent cases for each musculoskeletal disorder are presented as absolute numbers summed across the ten ASEAN countries for each age group; 95% uncertainty intervals (UIs) are not shown. ASEAN, Association of Southeast Asian Nations.

**Figure 2:**
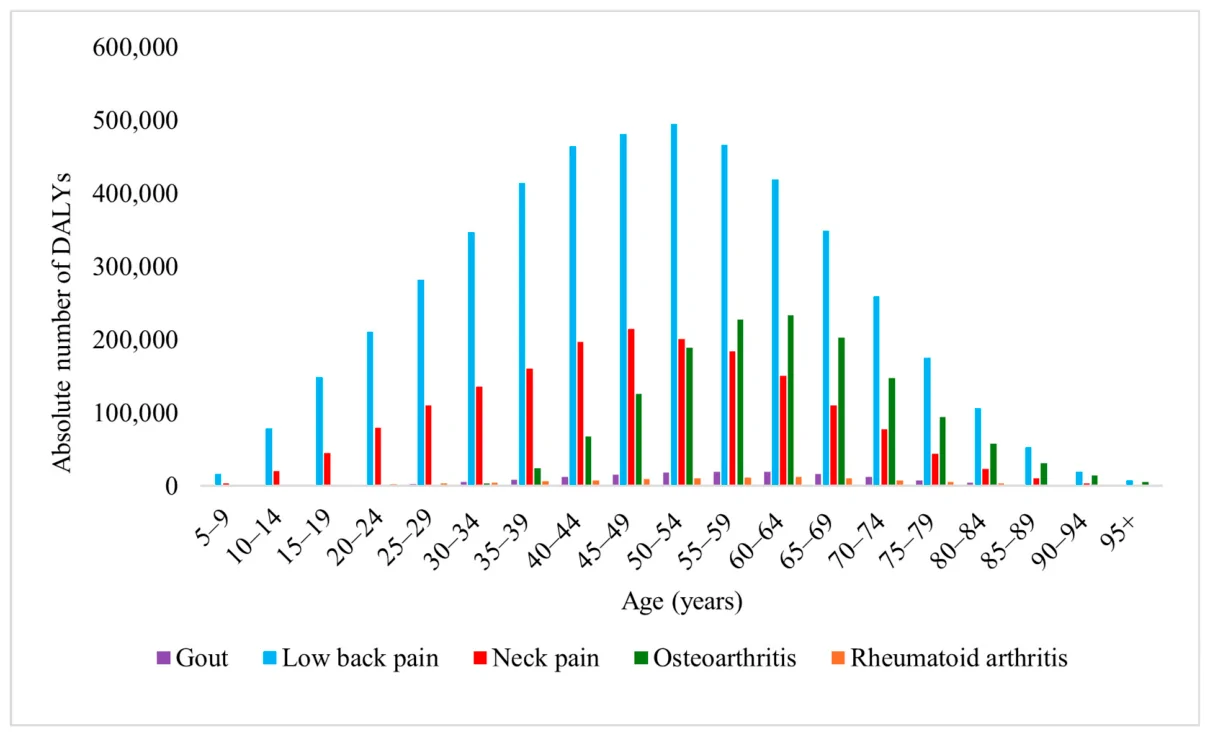
Age-specific absolute numbers of DALYs of the five musculoskeletal disorders across the ten ASEAN countries, 2023. DALYs for each musculoskeletal disorder are presented as absolute numbers summed across the ten ASEAN countries for each age group; 95% uncertainty intervals (UIs) are not shown. DALYs, disability-adjusted life years; ASEAN, Association of Southeast Asian Nations.

**Figure 3:**
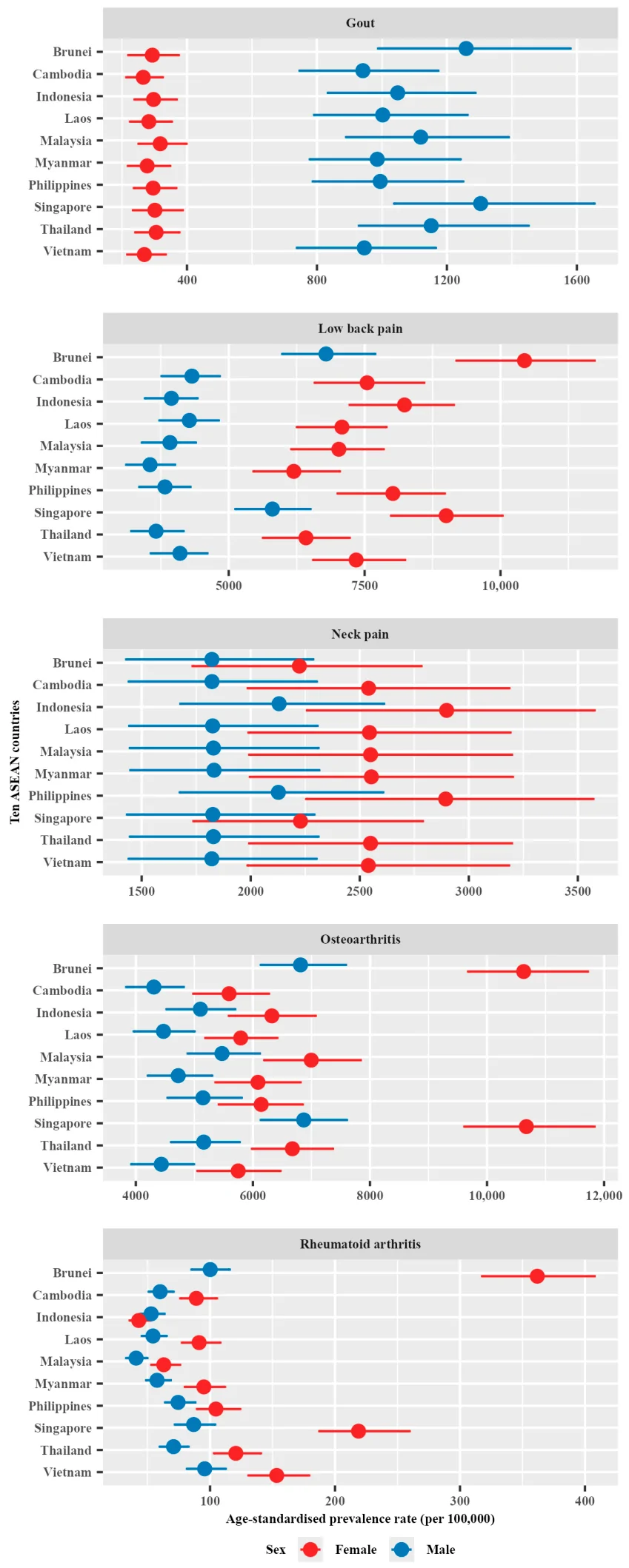
Country-specific age-standardised prevalence rates of five musculoskeletal disorders by sex, 2023. Dots represent age-standardised prevalence rates, and horizontal lines represent 95% UIs. From top to bottom, the five panels represent gout, low back pain, neck pain, osteoarthritis, and rheumatoid arthritis, respectively. Within each panel, the ten ASEAN countries are presented from top to bottom: Brunei, Cambodia, Indonesia, Laos, Malaysia, Myanmar, the Philippines, Singapore, Thailand, and Vietnam. UIs, uncertainty intervals.

**Figure 4:**
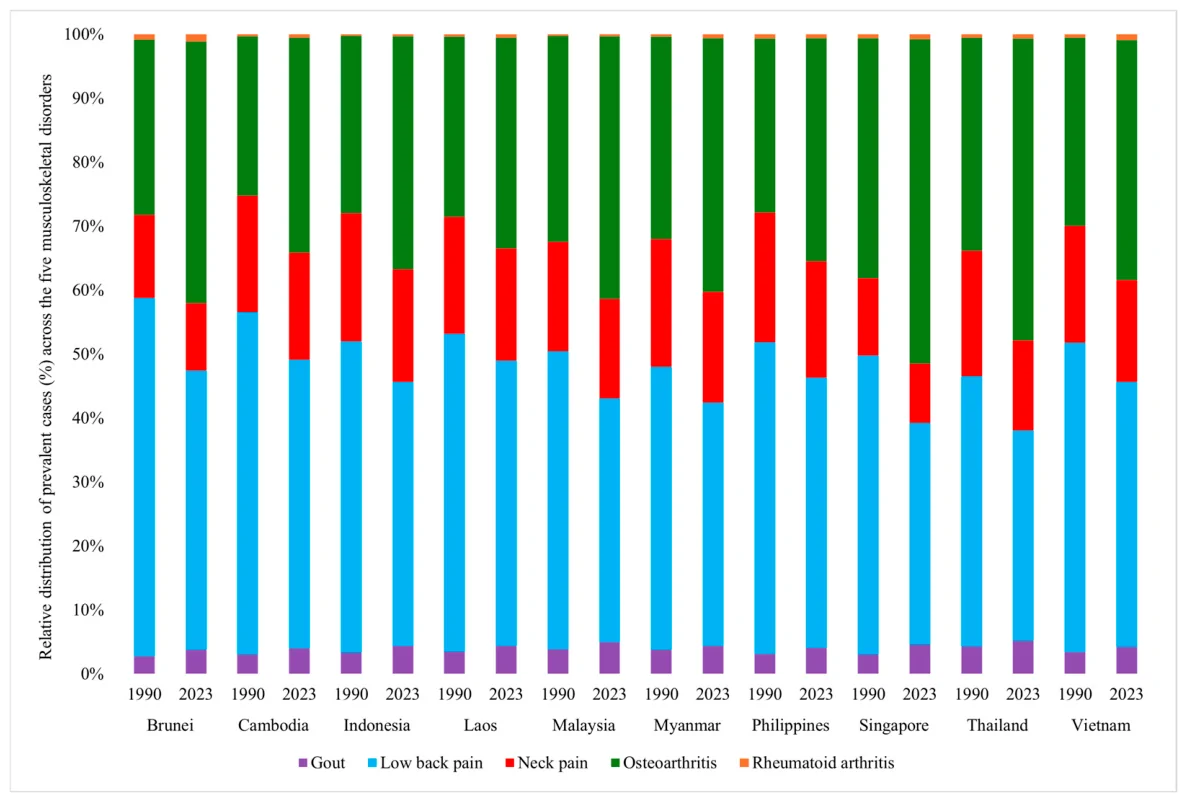
Relative distribution of prevalent cases (%) across the five musculoskeletal disorders in 1990 and 2023. Proportions are based on absolute numbers of prevalent cases for each musculoskeletal disorder relative to the total number of prevalent cases across the five musculoskeletal disorders within each country.

**Figure 5:**
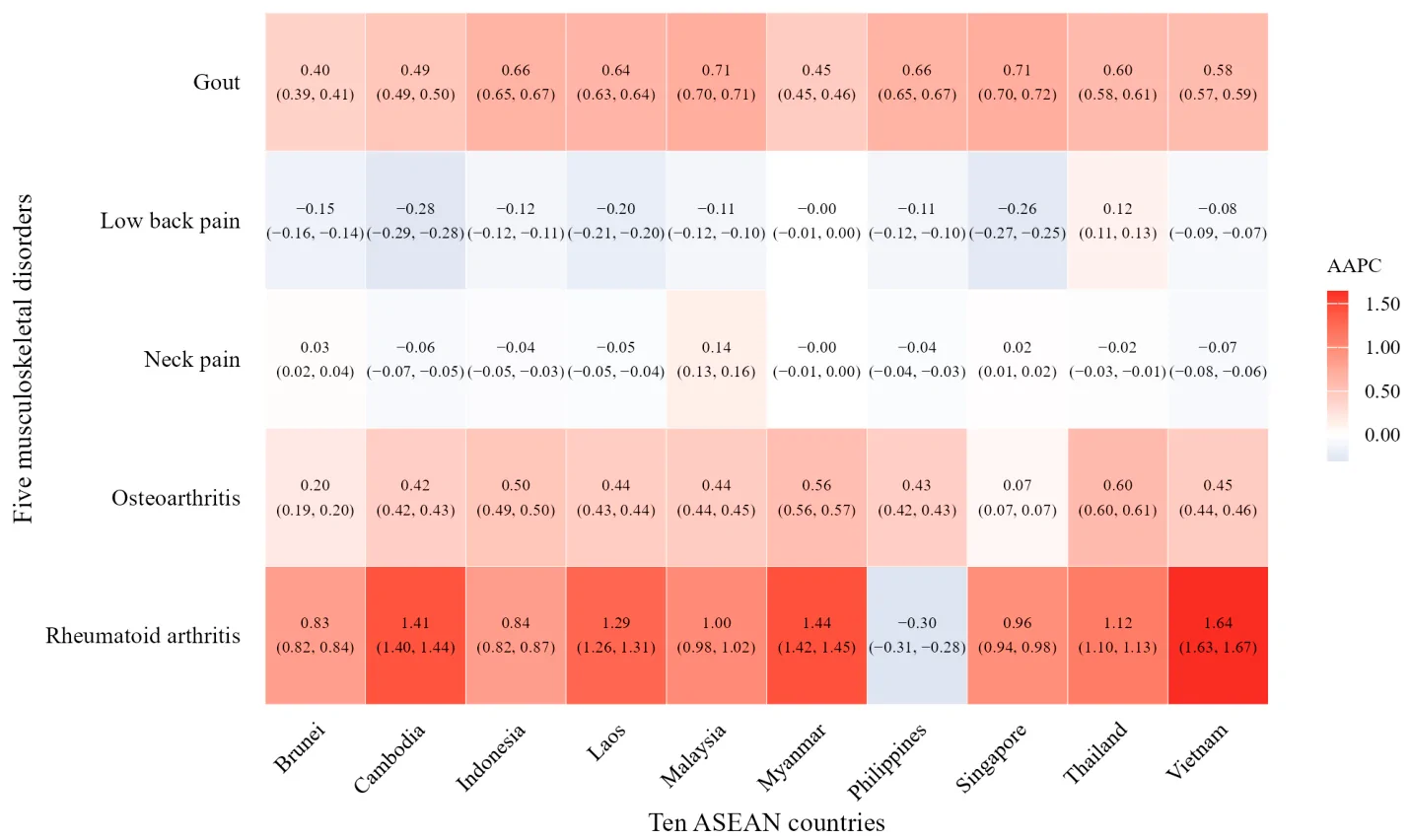
Country-specific AAPC in age-standardised prevalence rates of five musculoskeletal disorders, 1990–2023. Values within the tiles represent AAPC estimates and 95% CIs. Red indicates increasing trends, whereas blue indicates decreasing trends; colour intensity reflects the magnitude of change. AAPC was considered statistically significant if *p* < 0.05 and the 95% CI did not include zero. AAPC, average annual percent change; CIs, confidence intervals.

## Data Availability

All data used in this study were obtained from the Global Burden of Disease Study 2023 and are publicly available online via http://vizhub.healthdata.org/gbd-results/ (accessed on 9 December 2025).

## Supplementary Materials

The following supporting information can be downloaded at: https://www.mdpi.com/article/10.3390/jcm15197476/s1, Table S1. Country-specific age-standardised prevalence rates of five musculoskeletal disorders by sex, 2023; Table S2.1. Prevalence and DALYs of gout in 1990 and 2023, and percentage change from 1990 to 2023, globally and across the ten ASEAN countries; Table S2.2. Prevalence and DALYs of low back pain in 1990 and 2023, and percentage change from 1990 to 2023, globally and across the ten ASEAN countries; Table S2.3. Prevalence and DALYs of neck pain in 1990 and 2023, and percentage change from 1990 to 2023, globally and across the ten ASEAN countries; Table S2.4. Prevalence and DALYs of osteoarthritis in 1990 and 2023, and percentage change from 1990 to 2023, globally and across the ten ASEAN countries; Table S2.5. Prevalence and DALYs of rheumatoid arthritis in 1990 and 2023, and percentage change from 1990 to 2023, globally and across the ten ASEAN countries; Table S3. Relative distribution of prevalent cases (%) across the five musculoskeletal disorders in 1990 and 2023.
