# Supplementary material for: Burden of Five Musculoskeletal Disorders in ASEAN Countries, 1990–2023: A Global Burden of Disease Study 2023 Analysis
Source: J Clin Med. 2026 Sep 25;15(19):7476. doi: 10.3390/jcm15197476 (PMC13665756; doi:10.3390/jcm15197476)
Supplement: Supplementary file 1 [file jcm-15-07476-s001.zip › jcm-4528546-supplementary.pdf]

Title: Burden of Five Musculoskeletal Disorders in ASEAN Countries, 1990–2023: A Global Burden of Disease Study 2023 Analysis

Table of contents

Table S1: Country-specific age-standardised prevalence rates of five musculoskeletal disorders by sex, 2023 ………………………………...……………………..………. 2

Table S2.1: Prevalence and DALYs of gout in 1990 and 2023, and percentage change from 1990 – 2023, globally and across the ten ASEAN countries ……………...……. 3

Table S2.2: Prevalence and DALYs of low back pain in 1990 and 2023, and percentage change from 1990 to 2023, globally and across the ten ASEAN countries ……….. 4

Table S2.3: Prevalence and DALYs of neck pain in 1990 and 2023, and percentage change from 1990 to 2023, globally and across the ten ASEAN countries …………..... 5

Table S2.4: Prevalence and DALYs of osteoarthritis in 1990 and 2023, and percentage change from 1990 to 2023, globally and across the ten ASEAN countries …………. 6

Table S2.5: Prevalence and DALYs of rheumatoid arthritis in 1990 and 2023, and percentage change from 1990 to 2023, globally and across the ten ASEAN countries ……7

Table S3: Relative distribution of prevalent cases (%) across the five musculoskeletal disorders in 1990 and 2023…………………………....………………..…………….... 8

Table S1. Country–specific age–standardised prevalence rates of five musculoskeletal disorders by sex, 2023

Country	Sex	Gout	Low back pain	Neck pain	Osteoarthritis	Rheumatoid arthritis
Brunei	Female	293.32 (219.31 – 374.75)	10441.52 (9190.67 – 11730.58)	2223.13 (1733.31 – 2783.14)	10625.56 (9676.09 – 11723.47)	361.85 (317.69 – 407.69)
	Male	1259.28 (988.53 – 1580.31)	6790.06 (5984.72 – 7695.40)	1822.09 (1429.52 – 2286.45)	6813.57 (6136.20 – 7588.84)	100.13 (85.16 – 115.76)
Cambodia	Female	265.30 (213.17 – 325.51)	7546.03 (6582.61 – 8597.57)	2540.44 (1986.40 – 3185.98)	5592.73 (4981.34 – 6274.24)	88.99 (76.15 – 105.55)
	Male	941.38 (746.53 – 1173.55)	4323.23 (3764.81 – 4834.71)	1822.44 (1441.15 – 2302.77)	4308.03 (3837.19 – 4818.50)	60.03 (50.94 – 70.76)
Indonesia	Female	296.99 (238.53 – 368.35)	8234.26 (7225.80 – 9142.74)	2898.34 (2257.96 – 3576.64)	6324.29 (5589.39 – 7073.44)	42.92 (35.67 – 52.15)
	Male	1048.51 (833.64 – 1287.86)	3945.37 (3459.63 – 4425.53)	2130.16 (1677.64 – 2611.58)	5102.99 (4525.89 – 5698.39)	52.86 (44.20 – 63.59)
Laos	Female	282.59 (224.81 – 353.38)	7082.65 (6254.52 – 7902.59)	2544.59 (1989.57 – 3191.13)	5792.05 (5187.52 – 6417.47)	91.21 (77.49 – 108.21)
	Male	1002.40 (791.25 – 1263.53)	4275.86 (3726.26 – 4815.17)	1825.47 (1443.46 – 2306.36)	4472.55 (3965.81 – 5003.58)	54.31 (45.44 – 65.27)
Malaysia	Female	317.67 (250.83 – 398.74)	7025.83 (6152.40 – 7851.03)	2549.52 (1993.37 – 3198.00)	6996.46 (6194.53 – 7841.94)	62.98 (53.02 – 76.02)
	Male	1119.50 (889.84 – 1390.31)	3918.82 (3402.08 – 4393.97)	1829.07 (1446.31 – 2310.89)	5471.75 (4886.01 – 6118.11)	40.81 (32.97 – 49.86)
Myanmar	Female	277.46 (218.01 – 348.41)	6196.86 (5454.70 – 7043.89)	2553.17 (1996.23 – 3202.54)	6086.90 (5360.84 – 6814.56)	95.01 (79.81 – 111.96)
	Male	985.52 (778.11 – 1242.24)	3552.14 (3115.64 – 4010.96)	1831.74 (1448.44 – 2314.25)	4724.17 (4205.03 – 5304.98)	57.57 (48.89 – 68.50)
Philippines	Female	295.70 (236.41 – 367.01)	8020.37 (7004.07 – 8975.32)	2893.91 (2254.46 – 3571.23)	6141.73 (5417.90 – 6852.14)	104.68 (89.48 – 124.18)
	Male	994.73 (787.19 – 1250.08)	3826.48 (3355.63 – 4295.69)	2126.72 (1674.90 – 2607.38)	5147.52 (4542.12 – 5807.71)	74.42 (64.09 – 88.20)
Singapore	Female	301.19 (233.70 – 387.03)	9001.06 (7986.36 – 10038.36)	2228.17 (1737.26 – 2789.41)	10671.38 (9612.89 – 11836.84)	218.84 (187.47 – 259.70)
	Male	1303.92 (1037.65 – 1654.49)	5803.55 (5124.02 – 6503.40)	1826.25 (1432.80 – 2291.62)	6869.08 (6135.52 – 7605.37)	86.87 (71.86 – 104.10)
Thailand	Female	305.07 (240.78 – 376.74)	6420.74 (5629.83 – 7226.33)	2549.39 (1993.27 – 3197.84)	6674.51 (5984.51 – 7367.60)	120.61 (103.17 – 140.75)
	Male	1151.60 (929.31 – 1451.44)	3663.15 (3207.35 – 4169.88)	1828.98 (1446.24 – 2310.77)	5159.93 (4602.60 – 5773.05)	70.77 (59.80 – 82.74)
Vietnam	Female	268.74 (216.24 – 334.69)	7344.75 (6553.83 – 8244.92)	2539.32 (1985.51 – 3184.58)	5748.93 (5053.93 – 6470.91)	153.35 (130.77 – 179.32)
	Male	945.77 (738.55 – 1165.84)	4103.15 (3567.81 – 4606.89)	1821.61 (1440.50 – 2301.74)	4435.24 (3922.44 – 4993.93)	95.70 (81.54 – 112.57)

Data in parentheses are 95% uncertainty intervals.

Table S2.1. Prevalence and DALYs of gout in 1990 and 2023, and percentage change from 1990 to 2023, globally and across the ten ASEAN countries

	Prevalence (absolute number of cases)		Age–standardised Prevalence rate			Absolute number of DALYs		Age–standardised DALY rate		
	**1990**	**2023**	**1990**	**2023**	**Percent change, 1990 to 2023**	**1990**	**2023**	**1990**	**2023**	**Percent change, 1990 to 2023**
**Global**	22314749.94 (17875299.32 – 27944317.20)	58359797.62 (46265687.69 – 72970308.11)	536.03 (429.42 – 664.20)	642.56 (510.97 – 798.01)	19.87%	691821.17 (461847.56 – 985120.45)	1788634.60 (1196512.18 – 2540479.14)	16.48 (11.04 – 23.41)	19.71 (13.23 – 27.91)	19.60%
**Brunei**	915.58 (717.11 – 1161.16)	3707.93 (2887.24 – 4687.69)	687.22 (534.63 – 863.71)	781.57 (617.28 – 977.86)	13.73%	29.03 (19.05 – 43.33)	116.94 (77.74 – 171.66)	21.24 (14.33 – 30.88)	24.34 (16.28 – 35.65)	14.61%
**Cambodia**	24390.37 (19129.42 – 31068.84)	85708.65 (67563.11 – 107223.77)	482.58 (379.15 – 608.02)	567.42 (451.83 – 692.51)	17.58%	771.18 (491.17 – 1143.27)	2687.28 (1733.82 – 3857.86)	14.95 (9.45 – 22.02)	17.62 (11.69 – 25.48)	17.92%
**Indonesia**	606322.10 (481014.98 – 771327.54)	1923447.23 (1496348.93 – 2431145.20)	533.39 (426.25 – 657.52)	662.74 (526.60 – 817.68)	24.25%	19097.27 (12653.93 – 27580.30)	60706.84 (40592.27 – 88578.94)	16.47 (10.96 – 23.36)	20.66 (13.82 – 29.73)	25.45%
**Laos**	11999.87 (9576.59 – 15071.45)	36593.11 (28697.58 – 45784.31)	517.22 (409.02 – 637.77)	636.80 (503.71 – 797.97)	23.12%	378.73 (243.01 – 547.90)	1170.50 (771.31 – 1717.05)	16.11 (10.45 – 23.29)	20.08 (13.46 – 28.83)	24.62%
**Malaysia**	61589.09 (48760.28 – 76934.39)	250567.23 (199320.33 – 313242.61)	576.02 (455.82 – 711.30)	726.70 (582.31 – 899.65)	26.16%	1953.53 (1273.41 – 2884.32)	7875.65 (5138.84 – 11291.98)	17.91 (11.66 – 26.46)	22.69 (14.93 – 32.25)	26.68%
**Myanmar**	131077.00 (102519.97 – 169478.56)	317661.21 (248298.73 – 408666.01)	509.65 (403.28 – 638.18)	591.09 (467.03 – 742.23)	15.98%	4094.23 (2742.79 – 5917.49)	9945.13 (6569.72 – 14442.73)	15.67 (10.66 – 22.61)	18.35 (12.24 – 26.48)	17.10%
**Philippines**	175075.88 (139001.88 – 222056.36)	594935.68 (466036.80 – 761648.69)	504.55 (403.67 – 636.12)	623.70 (493.65 – 785.18)	23.61%	5543.53 (3625.10 – 7961.81)	18816.54 (12258.95 – 27125.98)	15.68 (10.35 – 22.27)	19.51 (12.87 – 28.16)	24.42%
**Singapore**	16246.15 (12943.72 – 20646.61)	74212.80 (58939.79 – 94871.90)	641.32 (506.06 – 799.12)	810.77 (650.07 – 1025.02)	26.42%	513.70 (336.63 – 746.74)	2324.69 (1527.75 – 3328.29)	20.01 (13.06 – 29.05)	25.47 (16.85 – 36.58)	27.29%
**Thailand**	237553.75 (192261.70 – 300011.61)	756641.48 (588912.39 – 962316.19)	577.43 (466.17 – 713.73)	698.09 (562.55 – 876.84)	20.90%	7484.73 (5011.28 – 10690.07)	23435.29 (16104.69 – 33785.08)	17.92 (12.24 – 25.23)	21.78 (14.67 – 31.40)	21.55%
**Vietnam**	205962.00 (163867.15 – 261340.84)	670366.98 (521364.39 – 846162.29)	479.06 (381.54 – 605.45)	578.00 (453.44 – 715.67)	20.65%	6487.31 (4414.53 – 9347.50)	21098.43 (14037.63 – 30653.46)	14.97 (10.00 – 21.59)	18.12 (12.14 – 26.26)	21.05%

Data in parentheses are 95% uncertainty intervals.

Table S2.2. Prevalence and DALYs of low back pain in 1990 and 2023, and percentage change from 1990 to 2023, globally and across the ten ASEAN countries

	Prevalence (absolute number of cases)		Age–standardised Prevalence rate			Absolute number of DALYs		Age–standardised DALY rate		
	1990	2023	1990	2023	Percent change, 1990 to 2023	1990	2023	1990	2023	Percent change, 1990 to 2023
**Global**	387587010.02 (343747657.69 – 435445076.29)	634889195.16 (556563431.71 – 706521600.05)	8390.77 (7382.43 – 9350.73)	7261.78 (6381.3 – 8066.93)	–13.46%	43237975.88 (30475204.02 – 58342560.10)	70423113.85 (49691193.02 – 95064380.82)	931.59 (657.17 – 1259.67)	805.94 (569.06 – 1,084.10)	–13.49%
**Brunei**	18537.48 (16232.44 – 21176.86)	42978.00 (37369.23 – 48388.76)	8995.75 (7971.52 – 10029.05)	8539.70 (7515.40 – 9506.14)	–5.07%	2114.8 (1492.13 – 2825.97)	4891.61 (3450.84 – 6588.17)	1014.98 (718.11 – 1372.54)	964.66 (681.87 – 1304.49)	–4.96%
**Cambodia**	432377.00 (382318.15 – 493728.30)	973348.09 (848519.86 – 1103387.04)	6688.39 (5847.69 – 7557.74)	6077.04 (5276.58 – 6836.62)	–9.14%	48455.63 (34124.5 – 65471.20)	109586.44 (77410.71 – 146568.16)	741.81 (525.43 – 998.10)	679.57 (482.94 – 906.84)	–8.39%
**Indonesia**	8806343.62 (7807184.43 – 9920654.78)	18235204.19 (15824456.93 – 20444228.06)	6381.63 (5574.04 – 7126.11)	6119.64 (5357.17 – 6812.60)	–4.11%	992362.01 (693855.38 – 1333936.99)	2063115.84 (1437646.78 – 2798726.35)	711.14 (499.05 – 957.52)	686.11 (481.29 – 929.73)	–3.52%
**Laos**	170689.75 (151517.92 – 192507.88)	371367.86 (327637.92 – 420673.30)	6091.87 (5380.61 – 6816.79)	5695.58 (5027.81 – 6385.61)	–6.51%	19192.53 (13500.19 – 25814.05)	42303.72 (29671.64 – 56942.79)	680.55 (486.28 – 915.22)	643.18 (457.26 – 867.42)	–5.49%
**Malaysia**	746282.63 (671426.94 – 826420.87)	1932543.22 (1680768.97 – 2166164.28)	5650.28 (5113.39 – 6182.17)	5429.37 (4739.20 – 6016.85)	–3.91%	84702.58 (59058.79 – 115043.27)	218283.53 (150504.65 – 295338.81)	634.74 (443.78 – 861.97)	609.87 (422.51 – 827.93)	–3.92%
**Myanmar**	1523820.48 (1346546.00 – 1739500.59)	2777049.66 (2425808.99 – 3150017.65)	5021.02 (4457.15 – 5628.12)	5003.55 (4375.13 – 5637.74)	–0.35%	170673.36 (122888.11 – 225067.22)	312116.13 (221983.5 – 421685.06)	556.92 (404.18 – 747.91)	558.93 (399.16 – 754.92)	0.36%
**Philippines**	2729291.98 (2433915.73 – 3069516.10)	6199719.35 (5430034.69 – 6954678.20)	6244.88 (5465.64 – 6959.54)	6008.74 (5258.41 – 6703.27)	–3.78%	308323.12 (215255.71 – 413119.26)	701704.64 (490143.48 – 948486.77)	698.85 (487.08 – 945.75)	674.69 (473.13 – 915.31)	–3.46%
**Singapore**	247721.81 (219618.46 – 278948.55)	560194.96 (490731.13 – 626238.12)	8070.67 (7119.20 – 8987.07)	7385.54 (6589.42 – 8226.41)	–8.49%	28289.95 (19902.56 – 37850.17)	63631.92 (45245.83 – 88539.39)	914.22 (641.01 – 1235.02)	839.20 (595.53 – 1142.31)	–8.21%
**Thailand**	2353011.73 (2094665.63 – 2672885.94)	4821170.38 (4197001.24 – 5432789.61)	4945.12 (4390.02 – 5527.95)	5127.44 (4517.64 – 5757.54)	3.69%	265638.34 (188439.53 – 356430.95)	539678.26 (383284.76 – 740543.13)	552.28 (392.90 – 750.48)	577.06 (404.97 – 772.43)	4.49%
**Vietnam**	2958339.20 (2644117.26 – 3274924.74)	6566963.24 (5759047.11 – 7415354.42)	6009.11 (5357.72 – 6595.38)	5827.41 (5168.58 – 6528.42)	–3.02%	332269.93 (235370.01 – 443645.69)	742645.24 (525400.69 – 998260.98)	671.77 (481.84 – 904.43)	656.80 (466.24 – 877.30)	–2.23%

Data in parentheses are 95% uncertainty intervals.

Table S2.3. Prevalence and DALYs of neck pain in 1990 and 2023, and percentage change from 1990 to 2023, globally and across the ten ASEAN countries

	Prevalence (absolute number of cases)		Age–standardised Prevalence rate			Absolute Number of DALYs		Age–standardised DALY rate		
	**1990**	**2023**	**1990**	**2023**	**Percent change, 1990 to 2023**	**1990**	**2023**	**1990**	**2023**	**Percent change, 1990 to 2023**
**Global**	114855172.60 (88959579.05 – 143093936.57)	209943592.48 (165947148.01 – 260901688.24)	2437.13 (1920.31 – 3010.27)	2410.37 (1903.82 – 2991.71)	–1.10%	11415480.08 (7671990.05 – 16869691.99)	20718828.49 (13811104.34 – 30127417.36)	240.88 (161.43 – 349.03)	238.3 (158.61 – 346.25)	–1.07%
**Brunei**	4299.22 (3152.58 – 5497.26)	10397.24 (7894.01 – 13285.75)	1999.88 (1546.54 – 2478.84)	2011.59 (1577.79 – 2513.01)	0.59%	436.75 (281.09 – 658.27)	1046.59 (688.43 – 1545.45)	200.29 (131.66 – 294.53)	201.82 (131.90 – 298.41)	0.76%
**Cambodia**	147077.94 (112421.85 – 185386.39)	360794.17 (275807.09 – 454049.19)	2257.28 (1762.21 – 2826.85)	2206.58 (1720.50 – 2759.72)	–2.25%	14628.04 (9590.32 – 21174.72)	35986.56 (24011.12 – 51793.62)	221.77 (146.71 – 317.41)	218.93 (144.54 – 313.17)	–1.28%
**Indonesia**	3627754.07 (2798411.15 – 4507224.88)	7785504.06 (6037896.78 – 9747452.29)	2555.28 (2013.69 – 3154.83)	2515.08 (1981.59 – 3103.51)	–1.57%	363485.12 (242302.82 – 545556.70)	779144.58 (507569.21 – 1163098.82)	252.99 (168.11 – 369.52)	250.44 (166.33 – 367.76)	–1.01%
**Laos**	62587.54 (47932.93 – 78401.14)	146426.09 (111732.92 – 185587.02)	2228.16 (1736.57 – 2786.09)	2186.90 (1706.30 – 2731.85)	–1.85%	6266.52 (4160.34 – 9161.83)	14744.88 (9754.23 – 21530.96)	221.00 (147.00 – 315.98)	218.50 (144.20 – 313.86)	–1.13%
**Malaysia**	273612.87 (222561.75 – 337918.22)	789523.14 (606184.22 – 990581.21)	2071.74 (1706.16 – 2516.82)	2175.92 (1700.83 – 2714.63)	5.03%	27542.76 (18590.27 – 39684.98)	78958.9 (52241.63 – 113823.81)	205.93 (138.89 – 288.97)	216.88 (143.41 – 314.35)	5.31%
**Myanmar**	688203.25 (525548.70 – 863138.34)	1264904.65 (978223.52 – 1601303.27)	2227.30 (1736.62 – 2783.79)	2222.84 (1733.43 – 2781.91)	–0.20%	68591.39 (45627.40 – 99423.77)	125876.81 (83954.3 – 182504.55)	219.69 (146.81 – 313.74)	220.33 (146.88 – 317.19)	0.29%
**Philippines**	1136342.27 (876335.64 – 1410516.76)	2676597.76 (2082123.84 – 3326094.65)	2552.69 (2010.57 – 3150.98)	2514.32 (1980.74 – 3102.15)	–1.50%	114234.48 (76639.05 – 172892.92)	268787.56 (176514.28 – 401986.17)	253.55 (168.73 – 372.25)	250.84 (166.47 – 369.01)	–1.07%
**Singapore**	63996.54 (47834.08 – 81355.95)	150935.29 (117099.48 – 190002.03)	2016.79 (1553.92 – 2500.52)	2021.63 (1584.57 – 2526.04)	0.24%	6490.77 (4202.25 – 9744.59)	15130.59 (9874.77 – 22574.97)	203.03 (132.65 – 300.03)	204.03 (133.00 – 304.87)	0.49%
**Thailand**	1092511.00 (830876.13 – 1381171.95)	2065112.64 (1638446.41 – 2577144.09)	2226.21 (1735.51 – 2781.44)	2208.18 (1721.32 – 2760.79)	–0.81%	109307.01 (72591.43 – 160447.35)	204003.94 (132920.6 – 295624.59)	220.57 (146.40 – 315.63)	220.00 (145.31 – 315.83)	–0.26%
**Vietnam**	1116371.65 (856890.04 – 1380220.34)	2524438.12 (1956784.09 – 3172206.24)	2249.23 (1754.22 – 2816.75)	2193.50 (1711.36 – 2741.07)	–2.48%	111657.35 (74120.03 – 159646.66)	252602.11 (164445.6 – 366210.92)	223.32 (147.62 – 317.64)	219.27 (143.16 – 316.26)	–1.82%

Data in parentheses are 95% uncertainty intervals.

Table S2.4. Prevalence and DALYs of osteoarthritis in 1990 and 2023, and percentage change from 1990 to 2023, globally and across the ten ASEAN countries

	Prevalence (absolute number of cases)		Age–standardised Prevalence rate			Absolute Number of DALYs		Age–standardised DALY rate		
	1990	2023	1990	2023	Percent change, 1990 to 2023	1990	2023	1990	2023	Percent change, 1990 to 2023
**Global**	256800033.59 (230579472.49 – 283197228.05)	635389531.38 (572406765.53 – 701619839.38)	6391.45 (5751.52 – 7042.09)	6909.25 (6229.49 – 7628.33)	8.10%	8954104.93 (4269872.47 – 19280378.67)	22357933.84 (10657089.20 – 48153655.80)	222.94 (106.62 – 478.15)	243.01 (115.75 – 523.53)	9%
**Brunei**	9079.74 (8191.91 – 10058.20)	40257.90 (36533.54 – 44553.92)	8202.90 (7390.62 – 9095.57)	8754.36 (7958.04 – 9656.31)	6.72%	326.67 (154.46 – 718.55)	1468.59 (695.17 – 3184.7)	297.25 (141.71 – 646.26)	320.95 (152.63 – 690.38)	7.97%
**Cambodia**	200759.97 (178270.21 – 223523.86)	724426.39 (647491.42 – 805940.98)	4381.36 (3895.08 – 4908.42)	5028.62 (4478.22 – 5624.42)	14.77%	6793.43 (3266.93 – 14496.09)	24909.82 (11857.89 – 53917.08)	147.64 (70.53 – 312.24)	172.72 (82.32 – 372.60)	16.99%
**Indonesia**	5013859.12 (4441317.36 – 5586419.25)	16105705.58 (14230917.50 – 17982316.01)	4870.78 (4323.01 – 5457.95)	5735.43 (5080.58 – 6402.20)	17.75%	171831.11 (81087.19 – 366803.87)	562729.69 (264558.49 – 1234270.31)	166.59 (79.25 – 353.12)	200.32 (94.58 – 435.49)	20.25%
**Laos**	96872.14 (86373.47 – 108504.25)	274777.36 (245855.56 – 304298.53)	4465.10 (3987.80 – 4995.20)	5146.56 (4588.86 – 5719.17)	15.26%	3303.57 (1563.47 – 7190.89)	9562.33 (4528.14 – 21041.02)	152.07 (71.71 – 329.01)	179.13 (85.63 – 391.87)	17.80%
**Malaysia**	515551.76 (456876.83 – 576450.02)	2081909.50 (1849161.30 – 2320402.63)	5389.07 (4781.85 – 5995.45)	6227.33 (5534.61 – 6937.78)	15.55%	17871.18 (8389.47 – 39310.86)	73296.24 (34618.12 – 162387.5)	186.68 (88.04 – 410.70)	219.16 (103.59 – 483.12)	17.40%
**Myanmar**	1088505.02 (965246.07 – 1203427.88)	2899532.96 (2559449.39 – 3258701.33)	4570.39 (4081.72 – 5094.19)	5492.67 (4859.13 – 6138.22)	20.18%	36992.87 (17625.31 – 77936.06)	100515.71 (47903.8 – 218865.5)	154.83 (74.18 – 326.72)	190.26 (90.63 – 414.36)	22.88%
**Philippines**	1523958.56 (1346964.42 – 1697962.84)	5116678.42 (4501420.42 – 5733985.61)	4945.35 (4377.64 – 5528.64)	5684.44 (5017.43 – 6350.77)	14.95%	52653.6 (24784.76 – 115322.09)	179451.77 (84045.65 – 395715.22)	170.72 (80.88 – 370.12)	199.41 (93.71 – 436.37)	16.81%
**Singapore**	198938.59 (179416.36 – 219575.01)	820339.28 (744916.18 – 909049.59)	8554.18 (7749.15 – 9421.67)	8757.31 (7954.10 – 9680.23)	2.37%	7256.96 (3397.77 – 15931.73)	30352.08 (14412.84 – 65692.37)	313.65 (148.06 – 683.97)	323.73 (153.49 – 701.18)	3.21%
**Thailand**	1852394.70 (1651467.73 – 2062614.43)	6918270.88 (6152289.33 – 7715929.57)	4903.06 (4355.46 – 5466.54)	5975.08 (5323.04 – 6634.56)	21.86%	63382.02 (30423.12 – 137952.93)	242391.33 (114827.41 – 519401.5)	167.52 (80.48 – 362.79)	209.18 (98.68 – 449.03)	24.87%
**Vietnam**	1797185.83 (1599383.13 – 1996596.23)	5945415.18 (5246388.48 – 6654095.43)	4457.92 (3976.27 – 4941.76)	5161.60 (4542.48 – 5776.33)	15.78%	61295.40 (29383.87 – 128032.32)	206591.81 (97602.58 – 454415.79)	151.87 (72.87 – 317.55)	179.31 (84.67 – 393.75)	18.06%

Data in parentheses are 95% uncertainty intervals.

Table S2.5. Prevalence and DALYs of rheumatoid arthritis in 1990 and 2023, and percentage change from 1990 to 2023, globally and across the ten ASEAN countries

	Prevalence (absolute number of cases)		Age–standardised Prevalence rate			Absolute Number of DALYs		Age–standardised DALY rate		
	1990	2023	1990	2023	Percent change, 1990 to 2023	1990	2023	1990	2023	Percent change, 1990 to 2023
**Global**	7976828.65 (7058904.37 – 9100200.90)	18477245.55 (16535214.21 – 21033687.19)	182.52 (163.32 – 207.23)	206.21 (184.46 – 235.10)	12.98%	1644672.94 (1268940.09 – 2021669.31)	3337270.07 (2500043.88 – 4210842.08)	38.55 (30.05 – 47.40)	37.2 (27.81 – 47.12)	–3.50%
**Brunei**	270.88 (228.29 – 322.39)	1138.90 (974.56 – 1304.72)	174.75 (152.40 – 200.82)	228.82 (199.56 – 258.37)	30.94%	58.66 (43.55 – 78.41)	217.26 (160.95 – 287.84)	43.58 (31.96 – 57.92)	45.57 (34.05 – 60.09)	4.58%
**Cambodia**	2932.90 (2422.07 – 3531.09)	11922.67 (10124.64 – 14231.46)	47.68 (40.22 – 56.61)	75.66 (64.94 – 89.59)	58.68%	683.38 (463.07 – 980.15)	2171.15 (1455.9 – 2914.18)	11.74 (8.15 – 16.74)	14.33 (9.76 – 19.54)	22.06%
**Indonesia**	48816.67 (39204.58 – 60875.87)	144914.87 (119591.01 – 177691.02)	36.17 (29.58 – 44.29)	47.62 (39.65 – 57.54)	31.67%	9958.36 (6725.29 – 13634.82)	27849.2 (18781.82 – 38777.67)	8.19 (5.45 – 11.04)	9.55 (6.49 – 13.49)	16.51%
**Laos**	1300.68 (1068.75 – 1577.89)	4604.08 (3830.18 – 5498.37)	48.12 (40.55 – 56.68)	72.93 (62.04 – 85.71)	51.55%	300.26 (208.46 – 425.31)	890.57 (600.51 – 1231.9)	11.96 (8.16 – 16.97)	15.12 (10.17 – 20.49)	26.46%
**Malaysia**	4681.93 (3812.98 – 5848.45)	18564.13 (15467.78 – 22356.71)	37.32 (30.67 – 45.93)	51.66 (43.29 – 61.84)	38.41%	785.77 (506.06 – 1260.57)	2887.6 (1881.11 – 4414.31)	6.62 (4.39 – 10.54)	8.17 (5.34 – 12.77)	23.43%
**Myanmar**	14599.66 (12074.86 – 17616.80)	43647.13 (36562.65 – 51483.83)	49.09 (41.32 – 57.86)	78.14 (66.01 – 91.24)	59.17%	3677.90 (2508.85 – 5333.59)	8613.53 (5905.23 – 11816.34)	13.65 (9.31 – 19.43)	16.17 (11.22 – 21.83)	18.45%
**Philippines**	40947.35 (34083.80 – 49178.58)	91052.94 (77263.28 – 109595.29)	99.36 (85.53 – 117.46)	89.68 (76.94 – 106.99)	–9.74%	7735.77 (5613.33 – 10391.49)	17272.74 (12188.22 – 23631.91)	20.55 (14.88 – 26.64)	18.28 (13.30 – 24.81)	–11.02%
**Singapore**	3213.09 (2717.98 – 3803.36)	12818.14 (10874.18 – 15276.95)	110.91 (94.45 – 129.48)	151.36 (128.04 – 179.18)	36.47%	506.10 (360.38 – 701.24)	1813.24 (1243.29 – 2590.26)	18.13 (13.24 – 24.47)	21.42 (14.29 – 30.96)	18.14%
**Thailand**	30987.51 (25849.66 – 36654.06)	98017.47 (84519.38 – 113621.26)	67.19 (57.39 – 78.18)	97.35 (83.02 – 114.46)	44.89%	6545.36 (4634.06 – 9307.26)	18913.26 (13840.65 – 26008.61)	15.32 (11.01 – 21.87)	18.30 (13.45 – 24.70)	19.43%
**Vietnam**	34918.97 (29075.95 – 41423.67)	144586.75 (123751.48 – 168923.70)	73.55 (61.67 – 86.46)	125.31 (107.71 – 146.43)	70.37%	6614.07 (4684.72 – 8792.88)	23476.32 (16173.72 – 31707.22)	14.42 (10.36 – 18.96)	20.60 (14.22 – 27.74)	42.87%

Data in parentheses are 95% uncertainty intervals.

Table S3. Relative distribution of prevalent cases (%) across the five musculoskeletal disorders in 1990 and 2023

Country	Gout		Low back pain		Neck pain		Osteoarthritis		Rheumatoid arthritis	
	1990	2023	1990	2023	1990	2023	1990	2023	1990	2023
**Brunei**	2.77	3.77	56.00	43.64	12.99	10.56	27.43	40.88	0.82	1.16
**Cambodia**	3.02	3.97	53.54	45.14	18.21	16.73	24.86	33.60	0.36	0.55
**Indonesia**	3.35	4.35	48.65	41.26	20.04	17.62	27.70	36.44	0.27	0.33
**Laos**	3.49	4.39	49.70	44.54	18.22	17.56	28.21	32.96	0.38	0.55
**Malaysia**	3.85	4.94	46.59	38.09	17.08	15.56	32.19	41.04	0.29	0.37
**Myanmar**	3.80	4.35	44.22	38.03	19.97	17.32	31.59	39.70	0.42	0.60
**Philippines**	3.12	4.05	48.69	42.24	20.27	18.23	27.19	34.86	0.73	0.62
**Singapore**	3.06	4.59	46.73	34.61	12.07	9.33	37.53	50.69	0.61	0.79
**Thailand**	4.27	5.16	42.27	32.89	19.63	14.09	33.28	47.19	0.56	0.67
**Vietnam**	3.37	4.23	48.40	41.43	18.26	15.93	29.40	37.51	0.57	0.91

Proportions are based on absolute numbers of prevalent cases for each musculoskeletal disorder relative to the total number of prevalent cases across the five musculoskeletal disorders within each country.
